# Parents’ intention to get vaccinated and to have their child vaccinated against COVID-19: cross-sectional analyses using data from the KUNO-Kids health study

**DOI:** 10.1007/s00431-021-04094-z

**Published:** 2021-05-17

**Authors:** Susanne Brandstetter, Merle M. Böhmer, Maja Pawellek, Birgit Seelbach-Göbel, Michael Melter, Michael Kabesch, Christian Apfelbacher, KUNO-Kids study group, Andreas Ambrosch, Petra Arndt, Andrea Baessler, Mark Berneburg, Stephan Böse-O’Reilly, Romuald Brunner, Wolfgang Buchalla, Sara Fill Malfertheiner, André Franke, Sebastian Häusler, Iris Heid, Caroline Herr, Wolfgang Högler, Sebastian Kerzel, Michael Koller, Michael Leitzmann, David Rothfuß, Wolfgang Rösch, Bianca Schaub, Bernhard H.F. Weber, Stephan Weidinger, Sven Wellmann

**Affiliations:** 1 University Children’s Hospital Regensburg (KUNO), Regensburg, Germany; 2 Member of the Research and Development Campus Regensburg (WECARE), Hospital St. Hedwig of the Order of St. John, Regensburg, Germany; 3 Bavarian Health and Food Safety Authority, Oberschleissheim, Magdeburg, Germany; 4 Institute of Social Medicine and Health Systems Research, Otto von Guericke University Magdeburg, Magdeburg, Germany; 5 University Department of Gynecology and Obstetrics, Hospital St. Hedwig of the Order of St. John, University Medical Center Regensburg, Regensburg, Germany

**Keywords:** COVID-19, Vaccination, Vaccination hesitancy, Parents

## Abstract

A COVID-19 vaccine can be an important key for mitigating the spread of the pandemic, provided that it is accepted by a sufficient proportion of the population. This study investigated parents’ intention to get vaccinated and to have one’s child vaccinated against COVID-19. In May 2020, 612 parents participating with their child in the KUNO-Kids health study completed an online survey. Multivariable logistic regression models were calculated to analyze predictors of intention to vaccinate. Fifty-eight percent of parents intended to get vaccinated against COVID-19, and 51% intended to have their child vaccinated. Significant predictors for the intention to get vaccinated and for having the child vaccinated included stronger parental confidence in one’s knowledge about prevention measures and lower beliefs that policy measures were exaggerated.

*Conclusion:* COVID-19 vaccination hesitancy was considerable in our sample of parents in Germany. However, our study revealed some potentially modifiable factors which should be addressed by a comprehensive and tailored communication and education strategy.

**Table:** 

**What is Known?** *• A COVID-19 vaccine can mitigate the spread of the pandemic.* *• Many parents are skeptical about vaccinations in general.*	
**What is New?** *• COVID-19 vaccination hesitancy was considerable in our sample of parents from Germany, not only for getting vaccinated but also for having the child vaccinated.* *• Negative parental attitudes regarding policy measures to contain the pandemic were associated with a lower intention to vaccinate.*

**Supplementary Information:**

The online version contains supplementary material available at 10.1007/s00431-021-04094-z.

## Introduction

The COVID-19 pandemic continues to threaten societies and their healthcare systems. Although safe and effective vaccines have been developed and approved for adults, major challenges remain, such as the provision of comprehensive access to vaccines and the reluctance of some people to get vaccinated.

In Western countries, a substantial proportion of the population is hesitant about vaccinations. For example, influenza vaccination uptake among pregnant women, healthcare workers, or the elderly is low in most European countries [7, 10]. Vaccination coverage is also insufficient in children. In Germany, despite many efforts at the end of the second year of life only 64% of children had received the second immunization against measles [11].

At the time of this study – considering this general vaccination hesitancy – it was questionable whether a sufficient proportion of people would intend to get vaccinated against COVID-19. On the one hand, the foreseeable fast development of COVID-19 vaccines was likely to elicit concerns. On the other hand, the pandemic attracted attention on an unprecedented scale, and people might be eager to protect themselves and others by getting vaccinated.

The aim of this study was to investigate the intention to get vaccinated and to have one’s child vaccinated against COVID-19, thus contributing to the emerging body of evidence on people’s vaccine acceptance. Using a large sample of parents who participate with their child in the KUNO-Kids health study prevalences and predictors of vaccination intention were analyzed.

## Methods

### Design

This study had a cross-sectional design. We used data collected in an online survey completed by parents participating in the KUNO-Kids health study between 5 and 28 May 2020. The KUNO-Kids health study is a multipurpose birth cohort study situated in Eastern Bavaria (Regensburg), Germany. Women are approached during pregnancy or immediately after delivery and invited to participate in the study. Recruitment started in 2015 and is still ongoing. It is envisaged to follow-up children into young adulthood [4].

### Sample

All participants in the KUNO-Kids study with a child between 1.5 and 5 years of age who had agreed to be contacted again were invited by mail to take part in the survey. One thousand and two hundred ninety-six families were contacted; 74 letters were not delivered because of invalid address.

### Measures

#### Outcomes

This study investigated two outcomes: parents’ intention to get vaccinated and to have their child vaccinated. (“If there was an effective vaccine against COVID-19, would you get vaccinated?,”… would you have your child vaccinated? -“yes”, “no”, “I don’t know”). Items were developed based on research on vaccinations against other infectious diseases. For the analyses, two categories were built (yes vs. no/I don’t know).

#### Predictor variables

Variables considered as predictors included general characteristics (age of child and mother, parents’ education (less than 10 years/10 years/more than 10 years of schooling), parents’ country of birth (Germany/other)) and variables related to COVID-19 (informed by the COSMO survey [3]). Participants are asked whether family members/friends had a confirmed SARS-CoV-2 infection (yes, severe illness/yes, mild symptoms/no) or belonged to a risk group (yes/no), how much confidence they had in their knowledge about which safety measures were suitable for preventing infections (7 point Likert scale, not confident at all – very confident), whether they were concerned about their health or their family’s health, whether they regularly sought information about the Corona crisis, how much they trusted in policy measures, and whether they considered policy measures to be exaggerated (all 5 point Likert scale, not at all – extremely) (see Table Supplement).

### Statistics

A multivariable logistic regression model was performed for each of the outcome variables. Predictor variables were entered into multivariable models if they were associated with the outcome variable in a univariable logistic regression analysis (criterion: *p* < .2). All analyses were performed using SPSS.24. Odds ratios (ORs) with corresponding 95% confidence intervals (CIs) were computed.

## Results

Six hundred twelve families took part in the survey (50.1% out of all families with valid addresses). Eighty percent of questionnaires were completed by mothers, 10% by fathers, and 10% by mothers and fathers together, respectively. Participants’ children were at average 3.4 years old (SD = 0.9), in 11.9% of families at least one parent was born outside Germany, and in 78.1% of families at least one parent had attended school for more than 10 years. 24.4% reported that a family member or friend had been infected with SARS-CoV-2.

Overall, 58% of parents intended to get vaccinated against COVID-19, and 51% intended to have their child vaccinated. In the univariable models, for all predictor variables, the direction and size of the effect estimates were quite similar for the intention to get vaccinated and for the intention to have the child vaccinated, respectively (see Table 1).

**Table 1:** Determinants of parents’ intention to get vaccinated and to vaccinate the child: univariable and multivariable logistic regression analyses

	Intention to get vaccinated						Intention to vaccinate the child					
	Univariable			Multivariable			Univariable			Multivariable		
	OR	95% CI	*p*	OR	95% CI	*p*	OR	95% CI	*p*	OR	95% CI	*p*
Child’s age (years)	1.03	0.86–1.23	*.767*				1.13	0.94–1.35	.191	1.16	0.91–1.36	.279
Mother’ s age (years)	1.06	1.02–1.11	*.006*	1.02	0.97–1.07	.509	1.04	1.00–1.09	.040	1.01	0.96–1.05	.919
Low educational level(< 10 years of schooling)	0.53	0.14–2.05	*.358*	0.98	0.23–4.23	.977	0.37	0.08–1.75	.209	0.59	0.11–3.06	.528
Medium educational (10 years of schooling)	Ref.						Ref.					
High educational level (university entrance level)	2.86	1.88–4.32	*< .001*	**2.70**	**1.70–4.28**	**< .001**	2.37	1.56–3.60	< .001	**1.99**	**1.26–3.34**	**.003**
Migration background (yes)	1.03	0.63–1.70	*.90*				0.97	0.59–1.58	.892			
No COVID-19 in family, friends	Ref.						Ref.					
COVID-19 with mild symptoms in family/friends	1.42	0.89–2.25	*.139*	1.13	0.68-1.88	.631	1.30	0.83-2.04	.249	1.09	0.67-1.77	0.74
COVID-19 with severe symptoms in family/friends	1.65	0.91–2.97	*.098*	1.42	0.73–2.76	.299	1.59	0.90–2.79	.110	1.35	0.72–2.54	.346
Risk group member in family, friends (yes)	0.79	0.50–1.25	*.317*				0.63	0.40-0.99	.047	**0.59**	**0.36–0.99**	**.044**
Concerns about own health (0–4)	1.17	0.97–1.42	*.097*	1.12	0.87–1.44	.361	1.05	0.88–1.27	.575			
Concerns about family health (0–4)	1.22	1.04–1.44	*.016*	1.08	0.87–1.34	.500	1.10	0.95–1.30	.202			
Confidence in one’s knowledge about safety measures (0–6)	1.23	1.09–1.39	*.001*	**1.21**	**1.05–1.39**	**.008**	1.28	1.13–1.45	< .001	**1.23**	**1.07–1.41**	**.003**
Trust in policy measures (0–4)	1.69	1.4–2.02	*< .001*	1.19	0.94–1.51	.152	1.68	1.40–2.02	< .001	1.20	0.95–1.50	.127
Perception that policy measures are exaggerated (0–4)	0.50	0.41–0.60	*< .001*	**0.58**	**0.46–0.73**	**< .001**	0.54	0.45–0.65	< .001	**0.60**	**0.49–0.76**	**< .001**
Regular information seeking about Corona pandemic (0–4)	1.51	1.28–1.79	*< .000*	1.20	0.98–1.46	.073	1.52	1.28–1.80	< .001	**1.22**	**1.00–1.48**	**.050**

Multivariable analysis: *N* = 600; Nagelkerke’s *R*^2^: .20; *OR* odds Ratio; *95% CI* 95% confidence interval; *p p*-value; *ref.* reference category; educational level of the higher educated parent; migration background if at least one parent was born not in Germany; bold: statistically significant (*p *< .05) in the multivariable analyses

In the multivariable models, a higher educational level (compared to a medium level) and stronger confidence in one’s knowledge about prevention measures were associated with higher intention to get vaccinated and to have the child vaccinated, while stronger beliefs that policy measures were exaggerated were associated with a lower intention for both outcomes. Moreover, regular information seeking about the COVID-19 pandemic increased the intention to vaccinate the child, while the consideration of family or friends as risk group members decreased this intention (see Table 1).

## Discussion

With only 58% of parents stating their intention to get vaccinated against COVID-19 and 51% with the intention to have their child vaccinated, respectively, vaccination hesitancy was considerable in our sample of families in Germany. Also other studies showed that people were skeptical about a future COVID-19 vaccine. According to the representative COSMO survey in May 2020, about 60% of adults in Germany intended to get vaccinated [3]. This number continued to decrease until the end of 2020 and rose again with the second wave of COVID-19 infections and the start of the vaccination campaign in Germany to about 70% (March 2021) [2]. It will be crucial for the ongoing vaccination campaign to motivate also people who are considered at low risk for severe courses of COVID-19, e.g., young and healthy adults.

Until now, only a few studies addressed parents’ intention to have their child vaccinated. In line with our results, a study from the UK with parents of children younger than 18 months found that they were more hesitant to have their child vaccinated than to get vaccinated themselves [1]. A multinational study [6] showed that parents were more willing to vaccinate their child when it was older. This suggests that particularly young children are considered to be more sensitive to possible side effects of vaccination. Other studies focused on the quality of the approval process for a COVID-19 vaccine for children: While Skjefte et al. found that a major reason for mothers’ refusal of a COVID-19 vaccination for their child was concerns about insufficient data collection during the approval process [12], an international survey revealed that more than 40% of parents would accept even shortcuts in order to fasten the approval process for children [5]. In any case, once a COVID-19 vaccine will have been approved for children, special emphasis will be needed to convince parents that it is safe to have their child vaccinated.

When it comes to predictors of parents’ intention to get vaccinated, our study yielded interesting results: the finding of a lower intention to have the child vaccinated if close ones are at risk for COVID-19 was unexpected. Maybe the attribution of a risk to others was accompanied by the perception that one’s own family might be less affected. Parents’ perception that political measures might be exaggerated was associated with lower intention for vaccination. Even before the COVID-19 pandemic, people’s trust in their governments was crucial: agreement with populist parties was associated with negative attitudes towards vaccination [8]. This finding – together with the significant effect of parents’ education, regular information seeking and their confidence in knowledge about safety measures – emphasizes the importance of communication and education in order to address vaccination hesitancy.

Our study has some limitations: The survey was developed ad hoc and administered rapidly so that there was no space for validation and/or pretesting. It has also to be acknowledged that the situation regarding the spread of the COVID-19 pandemic, the associated policy measures, and the vaccination campaign is changing fast. We captured participants’ views in May 2020, a period when it was still unclear whether and when vaccines would be available. Since then much research on the intention to get vaccinated against COVID-19 has been conducted. A rapid systematic review of 126 surveys from 31 countries found that many determinants of COVID-19 vaccination acceptance were universally relevant [9]. We also believe that associations between predictor variables and vaccination intention we found in our study might be a function of the prevalence of vaccination utilization in the population as being vaccinated will become the social norm.

## Conclusion

In May 2020, the intention to get vaccinated oneself or have the child vaccinated against COVID-19 was low in our sample of parents in Germany. Our findings on predictors of intention to get vaccinated could contribute to the development of a comprehensive and tailored communication and education strategy.

## Supplementary Information

ESM 1 (DOCX 14 kb)

## Abbreviations

CI: Confidence interval
COVID-19: Coronavirus disease 2019
OR: Odds ratio
Ref: Reference category
SARS-CoV-2: Severe acute respiratory syndrome coronavirus 2
SD: Standard deviation
