# Supplementary material for: Parents’ intention to get vaccinated and to have their child vaccinated against COVID-19: cross-sectional analyses using data from the KUNO-Kids health study
Source: Eur J Pediatr. 2021 May 17;180(11):3405–10. doi: 10.1007/s00431-021-04094-z (PMC8127511; doi:10.1007/s00431-021-04094-z)
Supplement: Supplementary file 1 — (DOCX 14 kb) [file 431_2021_4094_MOESM1_ESM.docx]

Table Supplement: Descriptive statistics of participant characteristics and predictor variables

|  | N |  |
| --- | --- | --- |
| **Sociodemographic Variables** |  |  |
| Child’s age (years), M (SD) | 612 | 3.36 (0.89) |
| Mother’ s age (years), M (SD) | 606 | 36.33 (3.92) |
| Educational level | 609 |  |
| Low educational level(< 10 years of schooling), N (%) |  | 12 (2.0) |
| Medium educational (10 years of schooling), N (%) |  | 119 (19.5) |
| High educational level (university entrance level), N (%) |  | 478 ( 78.5) |
| Migration background (yes), N (%) | 598 | 73 (12.2) |
| **COVID-19 related variables** |  |  |
| COVID-19 in family, friends | 610 |  |
| *“Is there or has there been at least one person with confirmed COVID-19 within the family and circle of acquaintances?”* |  |  |
| No COVID-19 in family, friends, N (%) |  | 461 (75.6) |
| COVID-19 with mild symptoms in family/friends, N (%) |  | 93 (15.2) |
| COVID-19 with severe symptoms in family/friends, N (%) |  | 56 (9.2) |
|  |  |  |
| Risk group member in family, friends (yes), % | 610 | 520 (85.2) |
| *“Does anyone in your family/friends belong to a risk group with regard to the Corona virus?” (yes, no)* |  |  |
|  |  |  |
| Concerns about own health (0-4), M (SD) | 610 | 1.24 (0.87) |
| *“How much are you concerned about your own health? – Not at all… Extremely” (5-point scale)* |  |  |
|  |  |  |
| Concerns about family health (0-4), M (SD) | 610 | 2.16 (1.00) |
| *“How much are your concerned about the health of your family? – Not at all… Extremely” (5-point scale)* |  |  |
|  |  |  |
| Confidence in one’s knowledge about safety measures (0-6), M (SD) | 610 | 4.11 (1.34) |
| *“How confident or uncertain do you feel about what protective measures are appropriate to avoid infection with the novel coronavirus?- Not confident at all… Very Confident” (7-point scale)* |  |  |
|  |  |  |
| Trust in policy measures (0-4), M (SD) | 610 | 2.62 (0.96) |
| *“I trust in the measures taken by the federal and state governments. – Not at all… Extremely” (5-point scale)* |  |  |
|  |  |  |
| Perception that policy measures are exaggerated (0-4), M (SD) | 610 | 0.69 (1.02) |
| *“I consider the ordered policy measures exaggerate”. – Not at all… Extremely” (5-point scale)* |  |  |
|  |  |  |
| Regular information seeking about Corona pandemic (0-4), M (SD) | 610 | 3.09 (0.99) |
| *“I inform myself regularly about the current developments in the Corona crisis. – Not at all… Extremely” (5-point scale)* |  |  |

Notes: M: mean; SD: standard deviation; educational level of the higher educated parent; migration background if at least one parent was born not in Germany.
For the purpose of this publication, questions were translated in English.
